# Variations in *Trim5α* and *Cyclophilin A* genes among HIV-1 elite controllers and non controllers in Uganda: a laboratory-based cross-sectional study

**DOI:** 10.1186/s12977-020-00527-z

**Published:** 2020-07-06

**Authors:** Sharon Bright Amanya, Brian Nyiro, Francis Waswa, Bonniface Obura, Rebecca Nakaziba, Eva Nabulime, Ashaba Fred Katabazi, Rose Nabatanzi, Alice Bayiyana, Gerald Mboowa, Alex Kayongo, Misaki Wayengera, Obondo J. Sande

**Affiliations:** 1 Faculty of Health Sciences, Lira University, Lira, Uganda; 2 Department of Immunology and Molecular Biology, Makerere University College of Health Sciences, Kampala, Uganda; 3 Center for AIDS Research (CFAR) Lab, Joint Clinical Research Center, Kampala, Uganda; 4 The African Center of Excellence in Bioinformatics and Data Intensive Sciences, the Infectious Diseases Institute, McKinnell Knowledge Centre, Makerere University, Kampala, Uganda; 5 Makerere University Lung Institute, Kampala, Uganda

## Abstract

**Background:**

Tripartite Motif Containing 5 alpha (TRIM5α), a restriction factor produced ubiquitously in cells and tissues of the body plays an important role in the immune response against HIV. TRIM5α targets the HIV capsid for proteosomal destruction. Cyclophilin A, an intracellular protein has also been reported to influence HIV infectivity in a cell-specific manner. Accordingly, variations in TRIM5α and Cyclophilin A genes have been documented to influence HIV-1 disease progression. However, these variations have not been documented among Elite controllers in Uganda and whether they play a role in viral suppression remains largely undocumented. Our study focused on identifying the variations in TRIM5α and Cyclophilin A genes among HIV-1 Elite controllers and non-controllers in Uganda.

**Results:**

From the sequence analysis, the rs10838525 G > A mutation in exon 2 of TRIM5α was only found among elite controllers (30%) while the rs3824949 in the 5′UTR was seen among 25% of the non-controllers. In the Cyclophilin A promoter, rs6850 was seen among 62.5% of the non-controllers and only among 10% elite controllers. Furthermore, rs17860048 in the Cyclophillin A promoter was predominantly seen among elite controllers (30%) and 12.5% non-controllers. From gene expression analysis, we noted that the respective genes were generally elevated among elite controllers, however, this difference was not statistically significant (*TRIM5α* p = 0.6095; *Cyclophilin* A p = 0.6389).

**Conclusion:**

Variations in TRIM5α and Cyclophillin A promoter may influence HIV viral suppression. The rs10838525 SNP in TRIM5α may contribute to viral suppression among HIV-1 elite controllers. The rs6850 in the cyclophillin A gene may be responsible for HIV-1 rapid progression among HIV-1 non-controllers. These SNPs should be investigated mechanistically to determine their precise role in HIV-1 viral suppression.

## Background

Currently, 36.7 million people are living with HIV of which 70% are from the WHO African region [1]. To date, there is no documented cure, rather, HIV infected individuals are enrolled in lifelong Anti-retroviral Treatment (ART). Whereas ART enables them to live long healthy lives [1], there are concerns such as; viral latency, drug side effects and, resistance associated with long-term ART [2]. This creates a need to study host immune factors, restriction factors that enable host cells to resist HIV replication.

Restriction factors, dominantly acting proteins that function in a cell-autonomous manner to suppress HIV viral replication at distinct stages have been reported to influence HIV susceptibility and disease progression [3]. These include; Tripartite Motif-containing 5α (TRIM5α), Apolipoprotein B messenger RNA editing enzyme catalytic polypeptide-like 3 (APOBEC3), Tetherin/bone marrow stromal cell antigen (BST2) [3], Myxovirus resistance protein 2 (MxB), and Sterile α motif domain-HD domain-containing protein 1(SAMDH1) [4]. TRIM5α, a member of the tripartite motif-containing family of proteins restricts HIV by interfering with viral capsid uncoating hence terminating downstream processes that facilitate HIV genome integration [5]. It is also implicated in the modulation of innate immune signaling via nuclear factor kappaB (NF-kB) and activator protein 1 (AP-1) leading to the production of inflammatory cytokines such as interleukin-2 (IL-2) & interferon-gamma (IFN-γ), along with various cell surface markers [6]. Recent studies have reported that polymorphism in the human TRIM5α gene affects susceptibility to and progression of HIV infection. For example, R136Q single nucleotide polymorphism has been associated with resistance to HIV [7] while the defective H43Y mutation is reported to increase progress in HIV infection within the population [8]. Another intracellullar protein, Cyclophilin A (CypA) has been reported to influence HIV infectivity in a cell specific manner [9]. Consequently, polymorphisms in CypA gene have also been documented to influence susceptibility to HIV-1 infection [10].

The presence of HIV elite controllers, individuals who maintain undetectable viral load for more than 5 years without anti-retroviral therapy is proof that there are unique genetic, immunologic and virologic mechanisms that are protective to these people and would, therefore, be critical in developing effective host-directed therapies. In Uganda, Elite controllers constitute 0.26% [11] of the 1300,000 adults living with HIV in Uganda [12]. Exploring variations in TRIM5α and CypA genes among HIV-1 elite controllers is therefore essential to identify protective mutations that can be used as target molecular markers for host-directed therapy and screening tools for targeted anti-HIV-1 therapy. In this study, we report on the variations in TRIM5α and CypA genes as well as their expression patterns among HIV-1 elite and non-controllers in Uganda.

## Results

### Participant characteristics

This was a cross-sectional study conducted among 18 HIV-1 chronically infected individuals. These included 10 elite controllers [HIV plasma viral load < 50 viral RNA (vRNA) copies ml^−1^] and 8 non-controllers (ART controlled) whose demographic characteristics are summarised in Table 1.

**Table 1:** Demographic and clinical characteristics of study participants

Age	Sex	CD4 count^a^	Duration in Care (Years)	VL	Months between VLs	BMI^b^
Elite controllers						
53	F	1245	10	Undetectable	8	33.9
38	F	919	9	Undetectable	12	18.9
36	F	1188	7	Undetectable	8	38.5
56	M	833	7	Undetectable	9	17.2
42	F	909	5	Undetectable	9	31.8
30	F	1050	5	Undetectable	10	29.3
37	F	728	6	Undetectable	9	23.9
40	F	994	10	Undetectable	9	32.3
41	M	778	9	Undetectable	12	25.2
37	F	1063	6	Undetectable	8	26.1
Non-controllers						
40	M	920	6	10,500	6	27.2
41	F	1192	6	2840	10	37.5
40	F	940	5	10,800	15	26.3
29	F	747	5	14,800	8	–
43	F	781	8	2310	8	32.7
38	F	589	5	75,100	10	21.4
42	F	1021	8	5250	6	21.3
41	F	852	10	2850	7	30.2

^a^Baseline CD4 at time of recruitment

^b^BMI denotes body mass index

### TRIM5α and Cyclophilin A gene variations

**Fig. 1:**
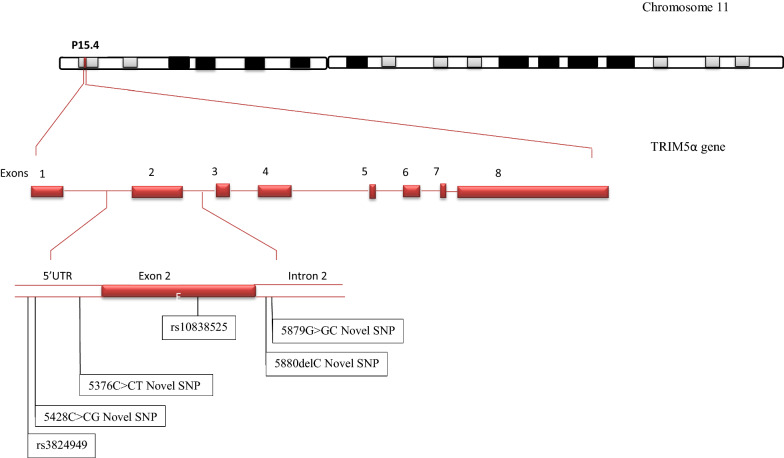
Gene map showing SNPs identified in human *TRIM5α* 5′UTR, exon 2 and intron 2

Considerable evidence suggests that variations in genes of intrinsic cellular defense against HIV influence HIV-1 disease progression [13, 14]. TRIM5α, one of the genes of intrinsic defense against HIV-1 and particularly it’s exon 2 that encodes for the ring domain that has E3 ubiquitin ligase activity and is important for the flexibility of TRIM proteins [15]. We sequenced the gene from the 5′UTR through exon 2 to intron 2. Previously stored PBMCs were thawed and then DNA extracted using Qiagen Blood Genomic DNA Kit (QIAamp DNA kit; Qiagen, Inc., Valencia, California, USA). The DNA was PCR amplified and then sequenced. Results indicate that rs10838525 single nucleotide polymorphisms (SNPs) were predominant among elite controllers (30%) while rs3824949 was more among non-controllers (25%) (Table 2; Fig. 1). Because SNPs in a coding region can affect protein function, we used the gomNAD browser to determine the effect of these mutations on protein function. The rs10838525 SNP in exon 2 was noted to cause R136Q amino acid change that is synonymous (Table 2; Fig. 1)

**Table 2:** TRIM 5a exon 2 SNPs among HIV-1 elite controllers and non-controllers

SNP	Chromosome Position	dbNo.	Aminoacid Change	Percentage (%)
Elite controllers (n = 10)				
5839G > GA	11:5701001	rs10838525	R136Q	30
5376C > CT	11:5701464	Novel SNP	5′UTR	10
Non controllers (n = 8)				
5431C > CG	11:5701409	rs3824949	5′UTR	25
5428C > CG	11:5701412	Novel SNP	5′UTR	12.5
5879G > GC	11:5700961	Novel SNP	Intron	12.5
5880delC	11:5700960	Novel SNP	Intron	12.5

**Fig. 2:**
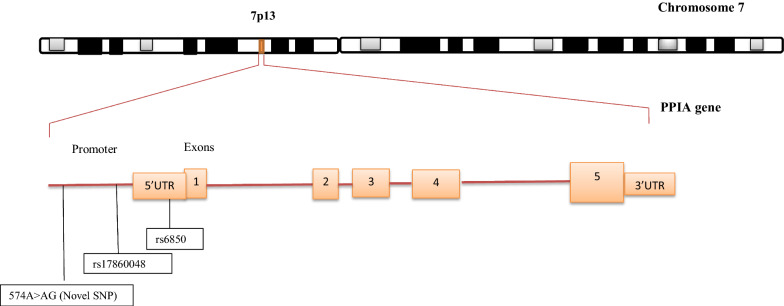
Gene map showing SNPs identified in the promoter region of human PPIA gene

Additionally, the promoter region for Peptidyl Prolyl Isomerase A (PPIA) gene which encodes for Cyclophilin A protein was also sequenced and SNPs characterized. We found that elite controllers had more rs17860048 SNP (30%) while rs6850 SNP dominated among non-controllers (62.5%) (Table 3; Fig. 2).

**Table 3:** Cyclophilin A SNPs among HIV-1 elite controllers and non-controllers

SNP	Chromosome Position	dbNo.	Percentage (%)
Elite controllers (n = 10)			
520C > CT	7:44836260	rs17860048	30
574A > AG	7:44836314	rs6850	10
435A > AC	7:44836175	Novel SNP	20
Non controllers (n = 8)			
574A > AG	7:44836314	rs6850	62.5
520C > CT	7:44836260	rs17860048	12.5
886dupG	7:44836626	Novel SNP	12.5

### TRIM5α and Cyclophilin A gene expression

**Fig. 3:**
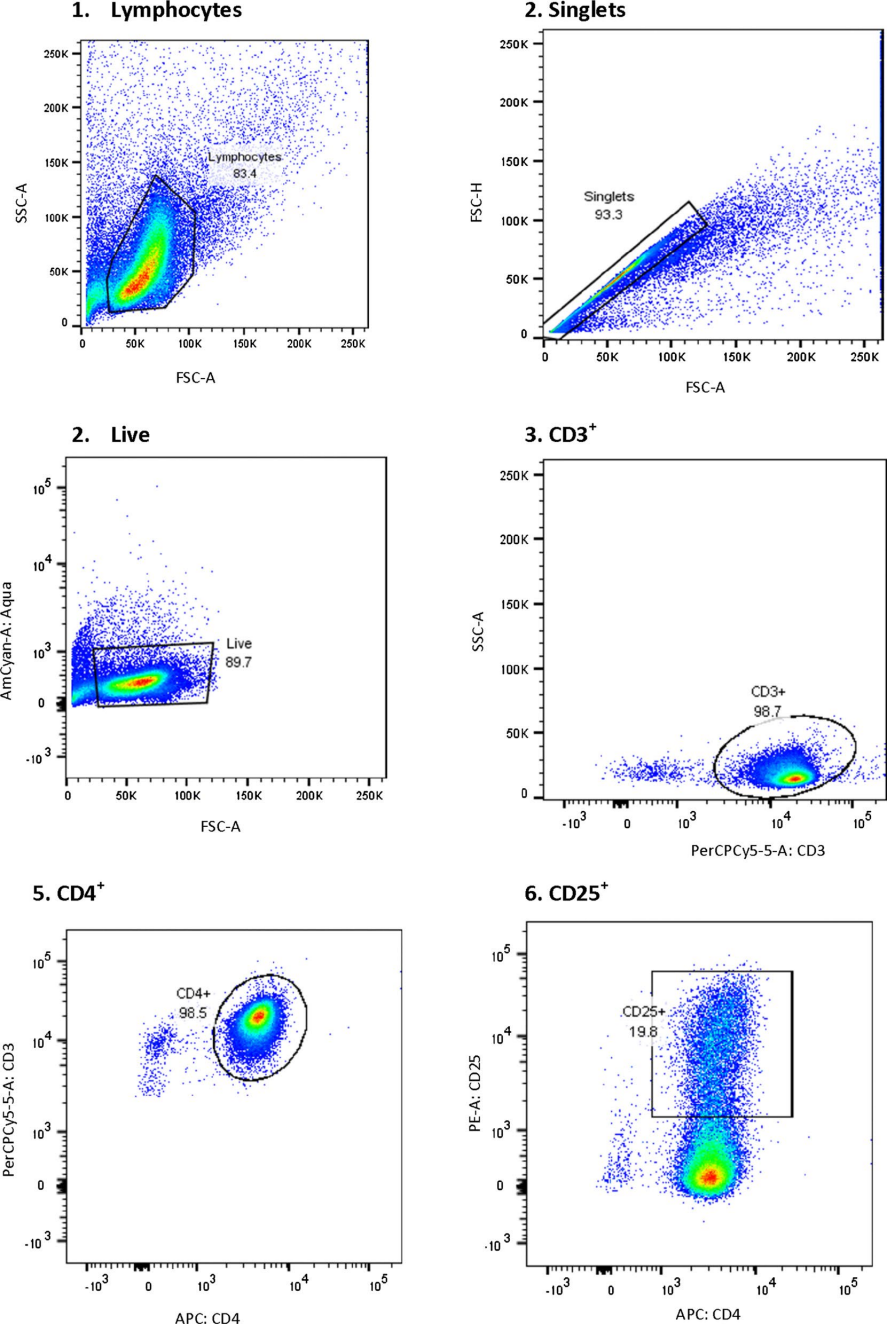
Cell activation prior to gene expression studies: A sequential gating strategy was used to confirm cell activation after 48 h of culture prior to gene expression studies

To determine the effect of these mutations on gene expression, CD4+T cells were isolated using human CD4^+^ T cell enrichment magnetic kit following the manufacturer’s instructions (StemCell Technologies, Vancouver, Canada). The CD4+T cells were assessed for purity by flow cytometry using the BD FACS CANTO II (BD Bioscience, Franklin Lakes, New Jersey, USA), and then stimulated with plate coated Anti-CD3 and soluble Anti-CD28 monoclonal antibodies for 48 h. The cells were confirmed for activation by flow cytometry prior to gene expression studies (Fig. 3).

**Fig. 4:**
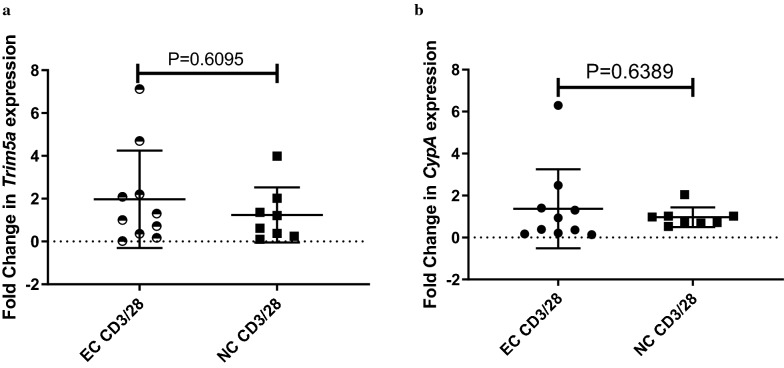
Graph A shows the difference in expression for TRIM5a gene is not statistically significant between elite controllers (EC) and non-controllers (NC) (p = 0.6095). Similarly, Graph B shows Cyclophillin A gene expression is more among elite controllers, but the difference is not statistically significant (p = 0.6369)

Total RNA was extracted using the Quick-RNA™ Whole Blood kit (Zymo Research, California, U.S.A) and mRNA levels of *TRIM5α* and *Cyclophilin A* was measured by RT qPCR. The respective genes were more expressed among HIV-1 elite controllers, however, the difference was not statistically significant (Fig. 4).

## Discussion

Our findings suggest that variations in TRIM5α and the regulatory region of Cyclophilin A genes influence HIV-1 viremic control and consequently HIV disease progression. We have identified rs10838525 SNP in exon 2 of TRIM5α which is predominated among HIV-1 elite controllers (30%) while rs3824949 in the 5′UTR of TRIM5α is concentrated among non-controllers (25%). The rs10838525 SNP in exon 2 results in the amino acid change from Arginine to Glutamine at codon 136 (R136Q). This has been reported to confer protection against HIV for high-risk individuals and slow progress of HIV disease for those infected [7, 13]. In the present study, we report a high frequency of rs10838525 among elite controllers (30%). These findings are comparable to the 32% documented among HIV negative healthy controls in a previous study conducted to identify the distribution of TRIM5α mutations among Brazilian HIV positive individuals and HIV negative healthy controls [16]. Taken together, these findings imply that the rs1083852 confers protection against HIV disease progression. In the present study, the rs3824949 in the 5′UTR of TRIM5α was seen more among HIV-1 non-controllers (25%). The 5′UTR region is known for anchoring binding sites for proteins that regulate translation in response to molecular signals [17], therefore a mutation in this region could affect TRIM5a gene expression, eventually influencing HIV disease progression. Sun et al. in their study among acutely and chronically HIV-infected patients showed that rs3824949GG genotype was associated with rapid disease progression while those with the CC genotype had reduced risk for rapid disease progression [18]. The CG genotype, however, had no significant association with rapid disease progression [18]. Similar findings were reported among HIV-1 positive Caucasian homosexual men enrolled in the Amsterdam cohort Studies (ACS) [14]. The role of rs3824949 genotype has also been observed in other diseases, with the GG genotype being associated with rapid antiretroviral treatment response compared to the CG and CC genotypes among Hepatitis C infected individuals [19, 20]. Since the CG genotype was seen among non-controllers in our study, it may not be of any significance in HIV disease progression.

In the current study, we found rs6850 SNP in the regulatory region of Peptidyl Prolyl Isomerase A (PPIA) gene that encodes for Cyclophilin A protein, to be more concentrated among HIV-1 non-controllers (62.5%). The presence of rs6850 SNP possibly increases Cyclophillin A expression which in turn increases HIV infectivity. Previous studies show that rs6850 is significantly associated with high HIV viral loads and lower CD4+T cell counts [21–23]. Moreover, the minor allele rs6850G found among 62.5% of the non-controllers has been previously reported to increase Cyclophillin A mRNA levels [21], thus implying that rs6850 SNP could increase HIV infectivity and disease progression by altering Cyclophillin A plasma levels. This is supported by a study among diabetes patients that demonstrated that rs6850 was associated with increased plasma levels of Cyclophillin A, and an increased likelihood of cardiovascular diseases among patients with or without diabetes [24]. Additionally, Rath et al. also reported that rs6850 was associated with recurrent myocardial infarction among patients with symptomatic coronary artery disease [25]. Taken together, these findings could imply that rs6850 increases Cyclophillin A protein expression that affects signaling and protein folding thus promoting susceptibility to various disease pathologies including HIV infectivity and disease progression among non-controllers. Another SNP, rs17860048 was found to be more prevalent among elite controllers; however, its role in HIV disease progression has not been reported.

Furthermore, we wanted to understand whether the expression of the respective genes varied between HIV-1 elite controllers and non-controllers. Our study findings show that TRIM5α and Cyclophilin A are highly expressed among elite controllers compared to the non-controllers. However, this difference was not statistically significant (TRIM5α p = 0.6095 and CypA p = 0.6389). These findings agree with those from previous studies (40). Vigneault et al. (2011) in their transcriptional profiling study of CD4^+^ T Cells among HIV-1 patients noted that gene transcripts known to be involved in intrinsic cellular defense against retroviruses, such as the TRIM, tetherin/BST2, cyclophilin A, and other genes were not differentially expressed among elite controllers compared to ART controlled HIV positive individuals [26]. Nonetheless, other studies have found a correlation between elevated expression of Cyp A and HIV disease progression [27]. These findings could mean that the viral suppression effect exhibited by elite controllers could be due to other mechanisms, not necessarily increased expression of the respective genes. However the small sample size in our study may have limited us in producing statistically significant results. Another limitation of this study was the gender imbalance, we cannot rule out the possible effect of gender on these findings. Future studies should consider a more gender balanced approach in investigating the role of variations in TRIM5a and Cylophillin A genes among HIV-1 elite controllers.

## Conclusion

In summary, our work reveals key SNPs within genes of intrinsic cellular defense against HIV that potentially play a role in HIV-1 viral suppression. Within the exon 2 of the TRIM5α gene, rs10838525 was only seen among elite controllers while rs6850 within the regulatory region of Cyclophillin A gene was seen predominantly among non–controllers (62.5%). These findings imply that variations in TRIM5α and cyclophilin A genes influence HIV-1 viral suppression. Furthermore, there was slightly higher TRIM5α and cyclophilin A gene expression among elite controllers as opposed to non-controllers although this difference was not statistically significant. This could imply that the elevated levels of genes involved in cellular intrinsic protective mechanisms against HIV may play a role in viral suppression exhibited by elite controllers and this effect needs to be investigated further with a large cohort of participants.

## Methods

### The aim, research design, and setting of the study

The study aimed at characterizing the variations in TRIM5α and CypA genes among Ugandan HIV-1 elite controllers and non-controllers.

A laboratory-based crossectional study was conducted utilizing PBMC samples from the Elite study cohort. The Elite study was conducted between 2016 and 2018 and its aim was to examine the role of host genes in T cell resistance to HIV among Elite and Viremic controllers in Uganda. The Elite study recruited participants from Makerere University Joint Aids Program (MJAP) ISS clinic.

The laboratory experiments were conducted at Makerere University College of Health Sciences, Molecular and Immunology Laboratories. Other assays were conducted at the Center For AIDS Research (CFAR) laboratory, Joint Clinical Research Center in Kampala, Uganda.

### Participant characteristics

The study utilized PBMC samples from two [2] patient groups, namely; a) HIV-1 elite controllers (undetectable viral load with > 5 years in care without ART) and b) non-controllers (HIV infected individuals well controlled on ART).

Elite controllers were selected basing on the following criteria; HIV infected individuals > 18 years old, have been confirmed to be HIV infected by HIV RNA PCR using Abbort real-time HIV-1 Assay (Abbott Molecular, USA), ART naïve for ≥ 5 years with CD4^+^ T cell count of ≥ 500 cells/ml, have a viral load of < 50copies/ml, have a hemoglobin concentration > 10 g/dl and are able to give written informed consent. Non-controllers were defined as HIV-1 infected individuals who are well controlled on ART. Being well controlled on ART meant CD4^+^ T cell count of > 500 cells/ml and no opportunistic infections. All those with active opportunistic infections e.g. Pneumocystis jiroveci pneumonia (PJP), Tuberculosis (TB), platelets < 50 and Bleeding disorders were excluded from the study.

### Laboratory methods

#### Treatment of PBMCs before storage

PBMCs isolated using Ficoll gradient centrifugation were washed with PBS and centrifuged at 1700 rpm for 5 min. The supernatant was discarded, and the pellet re-suspended in 40 ml PBS, and the wash step repeated twice. The cells were stained with trypan blue and counted using an automatic cell counter (Invitrogen, Carlsbad, California, USA). Those with viability ≥ 96% were prepared for storage. The cells were re-suspended in 1 ml of freeze media, then 0.5 ml of each sample aliquoted and stored in 2 cryovials. The cryovials were immediately placed in Mr. Frosty storage container (Thermo Fisher Scientific, Waltham, Massachusetts, USA), then stored overnight in a freezer at − 80 °C. The cryovials were transferred to liquid nitrogen for storage the following day until use.

#### Sample processing and thawing

PBMCs were retrieved from liquid nitrogen and immediately thawed in a water bath set at 37 °C. Thereafter, they were transferred into 10 ml of R-10 media and then centrifuged at 1500 rpm for 10 min. The supernatant was decanted, and the pellet resuspended in 5 ml R-10 media (10% FBS, 1% Pen-strep, 1% l-Glutamine, 1% Hepes Buffer, and RPMI) for counting. The cells were stained with trypan blue and counted using an automatic cell counter (Invitrogen, Carlsbad, California, USA). 1 ml of the sample was removed for DNA extraction.

#### CD4+T cell isolation

The thawed PBMCS were subjected to CD4^+^ T cell isolation using human CD4^+^ T cell enrichment magnetic kit following the manufacturer’s instructions (StemCell Technologies, Vancouver, Canada). The isolated CD4^+^ T cells were washed in 1 ml PBS, centrifuged at 1500 rpm for 10 min. These were resuspended in 2 ml R-10 media, stained for counting with trypan blue and then incubated at 37 °C on a 24 well plate for 2 h in a CO_2_ incubator. The cells were also stained for purity using anti-CD3, and anti-CD4 and ran on a BD FACS Canto II (BD Biosciences, Franklin Lakes, New Jersey, USA).

#### CD4+T cell Stimulation

A 96-well plate coated with 100 μl of 5 μg/ml of Anti-CD3 (eBioscience Clone CD28.2) was incubated at 37 °C for 2 h in a CO_2_ incubator. For negative control wells, 100 μl of PBS was added. After the 2 h incubation, the plate was bloated. In each well, 100,000 cells from the sample were added and topped up with R-10 media containing 5 μg/ml of anti-CD28 (eBioscience clone OKT3) to make 200 μl per well. For negative control wells, 110 μl of PBS was added. The plate was incubated at 37 °C for 48 h in a CO_2_ incubator.

### RNA extraction

RNA was extracted using Quick-RNA™ Whole Blood kit (Zymo Research, California, U.S.A) following the manufacturer’s instructions. The CD4^+^ T cell samples previously suspended in RNAlater (Sigma-Aldrich, St. Louis, Missouri, US) were centrifuged at 10.000 g for 1 min and then decanted. The pellet was re-suspended in 300 μl of DNA/RNA Shield™ then 30 μl PK digestion buffer and 15 μl Proteinase K added to the sample and mixed well. The mixture was incubated at 55 °C for 30 min. After incubation, the sample was vortexed and then centrifuged at 16,000 g for 2 min. The supernatant was transferred into RNase-free eppendoff tubes. To the supernatant, 350 μl of RNA recovery buffer was added and mixed well, transferred into a Zymo-Spin™ IIICG Column in a Collection Tube and centrifuged at 16,000*g* for 30 s. To the filtrate, 700 μl of 100% ethanol was added and mixed well. The mixture was transferred into a Zymo-Spin™ IC Column in a Collection Tube, centrifuged at 16,000*g* for 30 s and then the filtrate discarded. This was followed by DNase treatment to remove extra traces of DNA in the column. To achieve this, the column was washed with 400 μl RNA wash buffer and centrifuged at 16,000*g* for 30 s and thereafter the filtrate discarded. A Mixture of 5 μl DNase and 35 μl DNA digestion buffer was made and added directly to the column matrix. The column was incubated at room temperature for 15 min. After DNase treatment, 400 μl RNA prep buffer was added to the column and centrifuged at 16,000*g* for 30 s. The filtrate was discarded, and 700 μl RNA wash buffer added to the column and centrifuged at 16,000*g* for 30 s. The filtrate was discarded, 400 μl RNA wash buffer added and then centrifuged for 2 min at 16,000*g*. The column was then transferred into an RNase free eppendorf tube, thereafter, 15 μl DNase/RNase-free water added directly onto the column matrix to elute RNA. The eluted RNA was quantified by Qubit 4 Fluorometer (Invitrogen, Carlsbad, CA, USA). The RNA was then immediately stored at − 80 °C prior to downstream processes.

#### cDNA synthesis and reverse transcription PCR

Extracted RNA was subjected to cDNA synthesis and real-time PCR using QuantiTect Probe RT-PCR Kit (Qiagen Inc., Valencia, CA, USA) as described in the manufacturer’s instructions. A 50 μl reaction volume was used for the PCR. Primers and probes used were obtained from a previous study [28] and are summarized in Table 4. For each gene to be measured, separate master mix containing; a) 25 μl 2 × QuantiTect Probe RT-PCR Master Mix (HotStarTaq^®^ DNA Polymerase, QuantiTect Probe RT-PCR Buffer, dNTP mix, including dUTP, ROX™ passive reference dye, and MgCl2), b) 2 μl of each of the forward and reverse primers, c) 1 μl of the probe, d) 0.5 μl of the QuantiTect RT Mix, and e) 12 μl of the RNase free water. In every PCR tube, 42 μl of the master mix was added, and then 4 μl of RNA template added in 3 tubes containing master mix of the 3 respective genes namely; GAPDH (reference gene), Cyclophilin A (target gene), and TRIM5α (target gene). For each of the genes, a negative control was added in each of the experiments containing mastermix and PCR water but no RNA template added. The PCR tubes were loaded into the Rotor gene Q real-time PCR machine (Quiagen Inc, Valencia, California, USA) and PCR set using the following conditions; reverse transcription (cDNA synthesis) at 55 °C for 30 min, PCR initial activation at 95 °C for 15 min, followed by 45 cycles of denaturation at 94 °C for 15 s, and combined annealing and extension 60 °C for 60 s. Ct values for each gene were obtained and analyzed using the delta CT relative quantification method to determine the fold change in gene expression.

**Table 4:** Primers and probes used in reverse transcriptase PCR to quantify expression of TRIM5α, CypA and GAPDH

Protein	Primers and probes(Tamra)
TRIM5α F	5′-TGCCTCTGACACTGACTAAGAAGATG
TRIM5α R	5′-GGGCTAAGGACTCATTCATTGG
TRIM5α Probe	5′-(6-Fam)AAGCTTTTCAACAGCCTTTCTATATCATCGTGTGATA
CypA F	5′-GGCCGCGTCTCCTTTGA
CypA R	5′-AATCCTTTCTCTCCAGTGCTCAGA
Probe	(6-Fam)TGCAGACAAGGTCCCAAAGACAGCAG
GAPDH F	5′-ACCCCTGGCCAAGGTCATC
GAPDH R	5′-AGGGGCCATCCACAGTCTTC
Probe	5′-(6-Fam)AGGACTCATGACCACAGTCCATGCCA

### DNA extraction

DNA was extracted using the Qiagen Blood Genomic DNA Kit (QIAamp DNA kit; Qiagen, Inc., Valencia, California, USA) in accordance with the manufacturer’s instructions as used in the previous studies [29]. 20 μl of Qiagen Protease was pipetted into the bottom of a 1.5 ml microcentrifuge tube, then 200 μl sample added. 200 μl Buffer AL was then added to the sample and mixed by pulse-vortexing for 15 s. The mixture was incubated at 56 °C for 10 min and centrifuged to remove drops from the inside of the lid. 200 μl ethanol (96–100%) were added to the PBMCs and mixed again by pulse-vortexing for 15 s. After mixing, the tube was again centrifuged to remove drops from the inside of the lid. The reaction mixture was applied to the QIAamp Mini column, centrifuged for 6000*g* for 1 min and the filtrate discarded. The column was placed in a clean 2 ml collection tube. 500 μl of Buffer AW1 was then added to the QIAamp Mini column and centrifuged at 6000*g* for 1 min. The tube containing the filtrate was discarded and the column placed in a new clean collection tube. 500 μl Buffer AW2 was also added, centrifuged at 20,000*g* for 3 min and the tube containing filtrate discarded. The column was placed in a new collection tube, centrifuged at 20,000*g* for 1 min and the tube containing filtrate discarded. The QIAamp Mini column was then placed in a clean 1.5 ml microcentrifuge tube and 200 μl Buffer AE added. The mixture was incubated at room temperature for 1 min and then centrifuged at 6000*g* for 1 min to elute DNA. The extracted DNA was stored at − 80 °C prior to PCR amplification.

### PCR amplification

#### Exon 2 of TRIM5α gene

PCR amplification of TRIM5α gene (5′UTR, exon 2 & intron 2) was carried out with 35 cycles of denaturation at 94 °C for 30 s, annealing at 58 °C for 30 s, and extension at 68 °C for 45 s using SuperScript III platinum Taq polymerase (Invitrogen, Carlsbad, California, USA) in the presence of 2× reaction buffer, 5 mM MgCl with primers summarized in Table 5 as described in a similar study [13].

**Table 5:** Primers for amplification of of TRIM5α gene

Location	Primer
F	TGCAGGGATCTGTGAACAAG
R	CCATCTGGTCCCATTTTCTG

#### Cyclophillin A gene promoter

PCR amplification of the Cyclophilin A gene was carried out with 40 cycles of denaturing at 95 °C for 30 s, annealing at 65 °C for 45 s, and extension at 68 °C for 45 s using SuperScript III platinum Taq polymerase (Invitrogen, Carlsbad, California, USA) in the presence of 2X reaction buffer, 5 mM MgCl with primers summarized in Table 6 as described in a similar study [30].

**Table 6:** Primers used for amplification of Cyclophillin A promoter

Location	Primer
C1604G-F	GCACTGTCACTCTGGCGAAGTCGCAGAC
P4H-R	GCCGAGCACGTGCGTCGGACAGGAC

#### PCR Clean up

From all samples positive on gel electrophoresis that have a single band, 10 μl was aliquoted into a new PCR tube and 2 μl of ExosapIT reagent added. The tubes were then transferred into a thermocycler (Applied Biosystems, California, United States) and ran under the following conditions: 37 °C for 45 min, 80 °C for 45 min and held at 4 °C. Thereafter, PCR products were stored at − 20 °C prior to Sanger sequencing.

### Sanger sequencing

#### Cycle sequencing

Sequencing mastermix was prepared including 0.5 μl of big dye terminator, 1.75 μl of 5X sequencing buffer, 2.5 μl of primer, and 4.25 μl of water for the 10 μl reaction. 9 μl of the master mix was added into each plate well and 1 μl of the sample was then added. The plate was loaded in a SimpliAmp thermocycler (Applied Biosystems, California, United States), and ran under the following conditions; 96 °C for 1 min, then 30 cycles of 96 °C for 20 s, 50 °C for 20 s, and 60 °C for 4 min. Thereafter, the plate was held at 4 °C awaiting cleaning.

#### Clean up

Ethanol precipitation was done as follows. The 96-well sequencing reaction plate was removed from the SimpliAmp thermocycler and the plate centrifuged at 1000 rpm for one minute without cooling. To each well, 2.5 μl of 125 mM EDTA was added, followed by 1.0 μl 3 M Sodium Acetate pH 5.2 and then 30 μl of Absolute Ethanol to each well. The plate was sealed and vortexed briefly for 5 s, then incubated at room temperature for 30 min to precipitate the extension products. The plate was centrifuged at 3000 rpm for 60 min, at 4 °C. The plate cover was then removed, and the plate inverted on a paper towel placed in the microplate carrier assembly in the plate centrifuge. The plate was centrifuge at 500 rpm for one minute. 100 μl of 70% absolute Ethanol were added to each plate well and the plate heated at 90 °C for 1 min in a SimpliAmp thermocycler (Applied Biosystems, California, United States).

#### Electrophoresis

10 µl of 0.1 mM EDTA was added to each sample well and the plate sealed. The plate was vortexed for 5 s and then centrifuged at 1000 rpm for one minute without cooling to bring down the contents of the wells. The samples were then ready to run in the 3730xl DNA analyzer (Applied Biosystems, California, United States).

#### Data analysis

Data was entered in excel and exported to GraphPad prism 8 for analysis. CD4^+^ T cells were analyzed on an 8-laser FACS Canto II (BD Bioscience, Franklin Lakes, New Jersey, USA). Approximately 50,000 events were recorded per sample. In addition, antibody capture beads (BD Bioscience, Franklin Lakes, New Jersey, USA) were used for compensation and prepared individually by separate staining of all the antibodies used in the experiment. FlowJo X 10.6 (Treestar, Oregon, USA) was used for gating analysis. For mRNA quantification, relative quantification using the obtained CT value was done using the delta CT method. Statistical differences between the different groups were determined using the unpaired *t* test in Graph pad prism v8. Sequence analysis was done using Mutation Surveyor software to identify SNPs in the respective genes. Frequencies and percentages of the SNPs were determined. SNPs in the coding region were analysed using the gnomAD to determine the amino-acid change.

## Data Availability

The datasets used and/or analyzed during the current study are available from the corresponding author on reasonable request.

## Abbreviations

ART: Antiretroviral therapy
Cyclophilin A: CypA
DNA: Deoxyribonucleic acid
HIV: Human immunodeficiency virus
MJAP: Makerere University Joint Aids Program
PBMC: Peripheral blood mononuclear cells
PCR: Polymerase chain reaction
RNA: Ribonucleic acid
SNP: Single nucleotide polymorphism
TRIM5α: Tripartite Motif containing 5 alpha
WHO: World Health Organization
